# Neurofilament Light Chain in Serum and CSF as a Potential Biomarker for Primary Angiitis of the Central Nervous System

**DOI:** 10.3390/cells14130966

**Published:** 2025-06-24

**Authors:** Christina Krüger, Hans Pinnschmidt, Maximilian Wilmes, Justina Dargvainiene, Frank Leypoldt, Alexander Seiler, Daniela Berg, Tim Magnus, Milani Deb-Chatterji

**Affiliations:** 1 Department of Neurology, University Medical Center Hamburg-Eppendorf, 20246 Hamburg, Germany; m.wilmes@uke.de (M.W.); t.magnus@uke.de (T.M.); 2 Institute of Medical Biometry and Epidemiology, University Medical Center Hamburg-Eppendorf, 20246 Hamburg, Germany; h.pinnschmidt@uke.de; 3 Neuroimmunology Section, Institute of Clinical Chemistry, University Medical Center Schleswig-Holstein, 24105 Kiel, Germany; justina.dargvainiene@uksh.de (J.D.); frank.leypoldt@uksh.de (F.L.); 4 Department of Neurology, University Medical Center Schleswig-Holstein, 24105 Kiel, Germany; alexander.seiler@uksh.de (A.S.); daniela.berg@uksh.de (D.B.); milani.deb-chatterji@uksh.de (M.D.-C.)

**Keywords:** PACNS, NfL, vasculitis, biomarker, stroke, autoimmune diseases

## Abstract

Background: Primary angiitis of the central nervous system (PACNS) is a rare vasculitis affecting CNS blood vessels, posing diagnostic challenges due to the limited specificity of the established diagnostic tools. Neurofilament light chain (NfL) might be a promising biomarker in PACNS. Methods: NfL in serum and CSF was measured in 33 PACNS patients (25 active [aPACNS], 8 in remission [rPACNS]) enrolled between 2014 and 2022 and compared to controls (serum: *n* = 303; CSF: *n* = 68); Results: Serum NfL was significantly elevated in aPACNS (median: 45.77 pg/mL) versus rPACNS (6.68 pg/mL; *p* < 0.001) and healthy controls (6.05 pg/mL; *p* < 0.001). Similarly, CSF NfL was significantly elevated in aPACNS (median: 4914.58 pg/mL) compared to rPACNS (301.19 pg/mL; *p* = 0.002) and controls (262.83 pg/mL; *p* < 0.001). Serum and CSF NfL were significantly correlated (r = 0.90, *p* < 0.001). Additionally, an association between elevated NfL and ischemic lesions was observed (serum: r = 0.59, *p* = 0.006; CSF: r = 0.43, *p* = 0.032). A subgroup analysis excluding stroke patients still revealed elevated NfL in 90% (CSF) and 50% (serum), with diminishing discriminatory power with older age. Conclusions: NfL holds potential as a biomarker for PACNS, in particular in younger patients.

## 1. Introduction

Primary angiitis of the central nervous system (PACNS) is a rare form of vasculitis that causes inflammation of the blood vessels within the central nervous system [1]. The disease can be divided into small-vessel and large-/medium-vessel variants based on the size of the affected blood vessels [2,3]. PACNS often presents with nonspecific symptoms, such as headaches, progressive cognitive impairment, or seizures. With advancing disease progression, vascular inflammation may lead to vessel occlusion or rupture, resulting in ischemic or hemorrhagic infarctions [1,4]. Despite being a rare disease, PACNS accounts for a relevant proportion of juvenile strokes (approximately 2.2%) [5,6].

While brain biopsy remains the gold standard for diagnosing PACNS, diagnosis is often made through a combination of clinical presentation, magnetic resonance imaging (MRI), angiography (digital subtraction angiography [DSA] or magnetic resonance angiography [MRA]), and cerebrospinal fluid (CSF) analysis [7,8]. The reliance on less-invasive investigations reflects both the procedural risks of brain biopsy and its considerable false-negative rate. The histological sensitivity ranges from 53 to 74%, falling below 50% in some series, particularly in the medium-vessel variant [9,10]. Although a normal MRI virtually rules out cerebral vasculitis, its specificity remains limited. In large-vessel disease, black-blood vessel-wall imaging can demonstrate mural thickening or contrast enhancement, but the sensitivity and specificity of this finding have yet to be established [11]. Digital subtraction angiography (DSA) delineates caliber irregularities more clearly than MRA; however, once MRA has identified two or more stenoses, DSA seldom adds diagnostic value [12]. Moreover, angiographic abnormalities such as caliber jumps and segmental stenoses also occur in non-inflammatory vasculopathies such as reversible cerebral vasoconstriction syndrome or polyarteritis nodosa, yielding a low DSA specificity (14–60%) and a highly variable sensitivity (0–100%) [13].

Immunosuppressive treatment for PACNS typically involves cyclophosphamide or rituximab for induction therapy, followed by a maintenance treatment with, e.g., methotrexate or azathioprine [3]. Given that PACNS often follows a relapsing–remitting course, with fluctuating disease activity, continuous monitoring is essential for effective disease management [9]. Monitoring strategies include the assessment of clinical symptoms, repeated neurological examinations, neuroradiological imaging (MRI and MRA), and CSF analysis [14,15]. However, the nonspecific presentation and the low specificity of the common diagnostic tools frequently delay diagnosis of disease onset or relapse, which can postpone the adaptation or initiation of immunosuppressive therapy and consequently affect the clinical outcomes [16].

The identification of biomarkers that reliably reflect disease activity could greatly enhance both the diagnosis and monitoring of PACNS, potentially reducing the frequency of procedures such as biopsies, lumbar punctures, and MRI scans, thereby lowering associated risks and healthcare costs.

Neurofilaments, structural proteins that are essential for the axonal cytoskeleton, are released into the extracellular space following neuroaxonal damage, leading to elevated levels in both the blood and CSF [17,18]. The neurofilament light chain (NfL) subunit has emerged as a biomarker for axonal injury in various neurological diseases, such as multiple sclerosis (MS), amyotrophic lateral sclerosis (ALS), and traumatic brain injury (TBI) [15]. The ability to reflect the extent of neuroaxonal damage makes NfL a promising candidate for monitoring disease activity in PACNS, where neuroinflammation and vascular damage are key pathogenic features.

Thus, in this study, we aimed to investigate the feasibility of using NfL levels in serum and CSF as a potential biomarker for diagnosis, disease activity, and hence also treatment monitoring.

## 2. Materials and Methods

### 2.1. Patients and Healthy Controls

A total of 33 individuals with PACNS (*n* = 33) from the University Medical Center Hamburg-Eppendorf, Germany, were included in this retrospective analysis. Patients were enrolled and sampled between 2014 and 2022. Serum and CSF aliquots were stored at −80 °C and analyzed for NfL in April 2024. Twelve of the patients had biopsy-confirmed diagnoses (*n* = 12), while in twenty-one patients (*n* = 21), the diagnosis was based on clinical and radiological findings combined with the results of CSF analysis (“suspected PACNS”).

The cohort was divided into two subgroups based on disease activity. Patients were considered to have an active disease course (*n* = 25) if they were newly diagnosed or experienced a relapse. All patients in this group exhibited acute neurological symptoms, and/or brain MRI findings revealed new pathologies, such as ischemic strokes. Angiography (either DSA and/or MRA) was performed in 24 patients (*n* = 24) to identify new or progressive vessel irregularities, indicative of active medium-vessel vasculitis. CSF analysis was conducted during disease onset or relapse to confirm active CNS inflammation. Competing diagnoses, such as infections or non-vasculitic stroke etiologies were carefully ruled out by imaging, laboratory tests, and CSF analysis.

Patients were classified as being in remission (*n* = 8) at the time of sampling if they were asymptomatic or clinically stable. In four of these cases, lumbar punctures were performed to exclude subclinical CNS inflammation.

For the control group, *n* = 371 healthy individuals from the University Hospital Schleswig-Holstein were included. Serum controls (*n* = 303) were healthy laboratory volunteers with no neurological symptoms; no CSF was collected from this group. The CSF control subgroup (*n* = 68) consisted of individuals who underwent diagnostic lumbar puncture for suspected neurologic disease but were retained only if cerebrospinal fluid indices and brain MRI proved entirely unremarkable and no neurological disorder was diagnosed on follow-up.

The study was approved by the local ethics committee (Hamburg, Germany; PV5340), and written informed consent was obtained from all participants or their legal representatives.

### 2.2. NfL Measurement

All samples were measured in singlicate using the commercially available NF-light^®^ Advantage Plus assay (Quanterix, Billerica, MA, USA) on the fully automated HX Analyzer platform. To minimize batch effects, all samples were processed within a single run. High and low control samples were analyzed at the beginning and end of the run to ensure on-board reagent stability. NfL concentrations in serum and CSF were subsequently classified as normal or elevated based on age-adjusted reference ranges (Supplementary Table S1).

### 2.3. Statistical Analysis

Statistical analyses were performed using IBM SPSS Statistics (Version 29.0, IBM Corp., Armonk, NY, USA). Data are presented as median and interquartile range for non-normally distributed variables and as mean and standard deviation (SD) for normally distributed variables, unless otherwise specified. The Shapiro–Wilk test was used to assess the normality of data distribution. Pairwise Mann–Whitney U tests were used to compare groups for differences in NfL levels. All statistical tests were two-tailed, and a *p* value < 0.05 was considered statistically significant. Bonferroni correction was applied to account for multiple comparisons in the pairwise analyses. Since the NfL values showed a heavy right-skewed distribution, they were double-ln-transformed (ln(ln(x))) prior to further analyses with general linear models that assumed patient group, age, gender and the patient group x age interaction to be fixed effects. The adjusted R^2^ was used to evaluate the proportion of variance that was explained by the models. Model-estimated marginal means with 95% confidence intervals of NfL levels were computed for the patient groups and compared pairwise by least significant difference (LSD) tests. Back-transformed estimated marginal means are presented with their 95% confidence intervals.

## 3. Results

### 3.1. Patients with Active PACNS

Twenty-five patients (*n* = 25) had an active disease course of PACNS (median age 46 years, range 26–74 years, 44% male) and presented with acute neurological symptoms (Supplementary Table S2). Nine of them were diagnosed by histopathological evidence, and sixteen patients had suspected PACNS, diagnosed based on the results of MRI, angiography, and CSF. Fifteen patients were newly diagnosed, while ten had a relapse. In the group of patients with disease onset, two patients had been started on an immunosuppressive treatment prior to NfL sampling. In the relapse group, five of the ten patients suffered from a disease flare under immunosuppressive treatment. The CSF analysis revealed pleocytosis and/or elevated protein levels in 12 of 25 patients (48%), while oligoclonal bands (OCB) were found in 8 out of 25 patients (32%). At the time of sampling, recent ischemic lesions were present in 15 of 25 patients (60%), defined as acute or subacute infarcts on diffusion-weighted MRI.

### 3.2. Patients with PACNS in Remission

Eight patients (*n* = 8) were in remission at the time of NfL measurement (median age 49.5 years, range 30–72 years, 50% male, Supplementary Table S3). None of them showed clinical and/or imaging signs of disease activity. In three of the patients, the diagnosis was proven by biopsy, whereas five patients had a suspected PACNS. Four of the patients in the inactive group were treated with immunosuppressants at the time of the sampling, while immunosuppressive treatment was already tapered off in the others. CSF analysis was performed in four out of eight cases. None of the patients exhibited pleocytosis or elevated protein levels, and none of them showed positive OCB. Additionally, five of the eight patients underwent brain MRI at the time of sampling, none of which revealed new lesions.

### 3.3. Control Group

NfL in CSF was available for *n* = 68 controls (median age 40 years, range 16–85 years, 27.9% male), while serum NfL values were obtained from *n* = 303 controls (median age 43 years, range 18–71 years, 59.7% male).

### 3.4. NfL Levels in Serum

**Figure 1:**
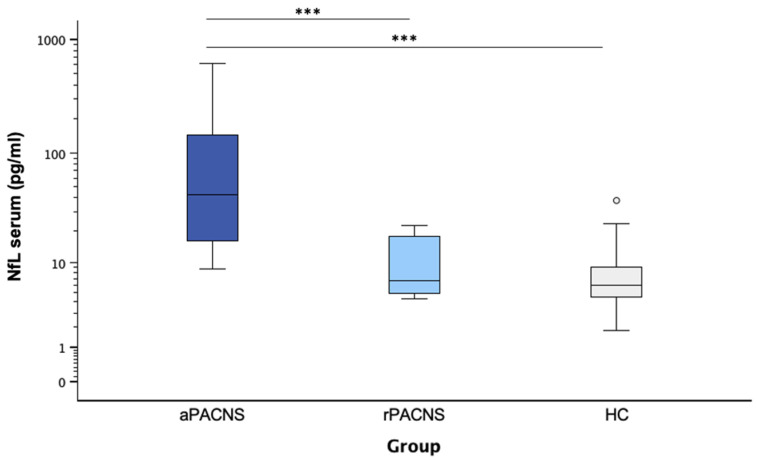
Distribution of serum NfL values in the present study population. Boxplot diagram showing the NfL values according to the group of patients included in the study, open circles indicate outliers. NfL in serum was significantly higher in aPACNS (*n* = 20) patients compared with both rPACNS (*n* = 8) patients and controls (*n* = 303). Pairwise Mann–Whitney U tests were used to compare groups for differences in NfL levels. All statistical tests were two-tailed, and a *p* value < 0.05 was considered statistically significant. Bonferroni correction was applied to account for multiple comparisons in the pairwise analyses. The asterisks reflect the level of significance. *** *p* ≤ 0.001. Abbreviations: aPACNS: active PACNS; NfL: neurofilament light chain; rPACNS: PACNS in remission.

Serum NfL levels (Figure 1) were significantly higher in active PACNS patients (median: 45.77 pg/mL; range: 8.76–617.48 pg/mL) than in the remission group (median: 6.68 pg/mL; range: 4.36–22.50 pg/mL; *p* = 0.001), as well as the healthy controls (median: 6.05 pg/mL; range: 1.81–37.73 pg/mL; *p* < 0.001). The difference between the remission group and healthy controls was not significant (*p* = 0.287). Elevated serum NfL levels were found in 70% (*n* = 14) of the active PACNS patients, while none of the rPACNS patients and 1.3% (*n* = 4) of the healthy controls exhibited elevated NfL levels.

### 3.5. NfL Levels in CSF

**Figure 2:**
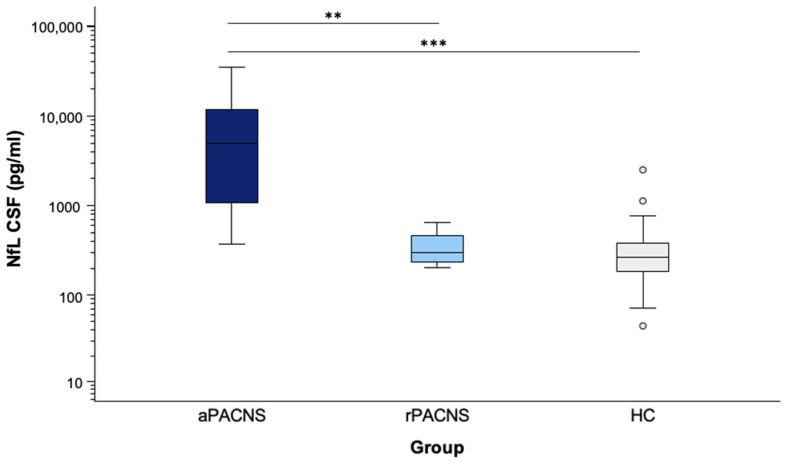
The distribution of CSF NfL values in the present study population. Boxplot diagram showing the NfL values according to the group of patients included in the study, open circles indicate outliers. NfL was significantly higher in aPACNS (*n* = 25) patients compared with both rPACNS patients (*n* = 4) and controls (*n* = 68). The asterisks reflect the level of significance. ** *p* < 0.01, *** *p* ≤ 0.001. Pairwise Mann–Whitney U tests were used to compare groups for differences in NfL levels. All statistical tests were two-tailed, and a *p* value < 0.05 was considered statistically significant. Bonferroni correction was applied to account for multiple comparisons in the pairwise analyses. Abbreviations: aPACNS: active PACNS; NfL: neurofilament light chain; rPACNS: PACNS in remission; CSF: cerebrospinal fluid.

Similarly, the CSF NfL levels (Figure 2) were significantly higher in active PACNS patients (median: 4914.58 pg/mL; range: 372.17–35,170.34 pg/mL) compared to both the remission group (median: 301.19 pg/mL; range: 201.30–647.95 pg/mL; *p* = 0.004) and healthy controls (median: 262.83 pg/mL; range: 44.80–2507 pg/mL; *p* < 0.001). No significant difference was observed between the remission group and healthy controls (*p* = 0.476). Elevated CSF NfL levels were present in 96% (*n* = 24) of active PACNS patients, while none of the rPACNS patients and 5.9% (*n* = 8) of healthy controls showed increased NfL levels.

### 3.6. Association Between Serum/CSF NfL Levels and Clinical Parameters in aPACNS

In patients with aPACNS, a highly significant positive correlation was observed between the serum and CSF NfL levels (r = 0.90; *p* < 0.001) (see Supplementary Figure S1). Notably, biopsy-proven PACNS was negatively associated with NfL concentrations (serum: r = −0.706, *p* < 0.001; CSF: r = −0.428, *p* = 0.033). Furthermore, NfL levels were significantly elevated in the presence of ischemic lesions (serum: r = 0.590, *p* = 0.006; CSF: r = 0.430, *p* = 0.032). In contrast, no significant correlations were found between NfL levels in aPACNS and other clinical or paraclinical parameters, including gender, CSF white cell count or total protein, contrast enhancement on imaging, or renal function (all *p* > 0.05).

### 3.7. PACNS Patients Without Ischemic Stroke

Since the results of a linear model analysis suggested that recent ischemic stroke might be a confounder for elevated NfL levels, we performed a subgroup analysis using age-dependent NfL cut-offs, comparing patients from the active PACNS group with (*n* = 15) and without (*n* = 10) recent stroke. Among the 10 patients without recent stroke, 9 had elevated NfL in the CSF (90%), whereas only 5 showed elevated serum NfL (50%). Of the 15 patients with stroke, all demonstrated elevated CSF NfL (100%). In the 10 serum samples available from aPACNS patients with recent stroke, 9 showed elevated serum NfL (60%).

Age-adjusted linear model analyses of patients without stroke indicated significant age x group interactions with respect to NfL levels (*p* < 0.05) (Supplementary Table S4). Thus, young patients with active PACNS had significantly higher CSF NfL and NfL serum levels than patients of the other two groups. For patients under 65, CSF NfL was significantly higher in active PACNS compared with both rPACNS (*p* = 0.048) and healthy controls (*p* = 0.003). In patients over 70, CSF NfL remained significantly higher in active PACNS compared with healthy controls (*p* = 0.044), but not compared to rPACNS (*p* = 0.130). Regarding serum NfL, significant differences were observed up to 60 years of age for both comparisons (active vs. inactive PACNS, *p* = 0.003; active vs. healthy controls, *p* < 0.001). In our patients over 65 years, the comparison between active PACNS and healthy controls remained significant (*p* = 0.008), but not for rPACNS (*p* = 0.199). In patients over 70 years of age, no significant differences were detected.

## 4. Discussion

PACNS is a rare and heterogeneous disease that often presents with nonspecific neurological symptoms. Diagnostic confirmation and disease monitoring currently depend on invasive procedures such as brain biopsy, lumbar puncture, or repeated neuroimaging. These approaches pose inherent risks, are often of limited specificity, and carry substantial costs. Consequently, reliable biomarkers that support both the diagnosis of PACNS and the assessment of disease activity would provide a less invasive, faster, and more accurate means of guiding clinical decisions. In this analysis, we evaluated the role of NfL as a biomarker in PACNS. NfL concentrations differed significantly between aPACNS patients, rPACNS patients, and healthy controls, indicating that NfL levels reflect disease activity in PACNS. Importantly, elevated NfL levels persisted even after excluding patients with recent ischemic stroke, with diminishing discriminatory power in older patients. While serum and CSF NfL levels were strongly correlated, measuring NfL in CSF proved more sensitive overall. Our findings demonstrate that NfL might be a potential biomarker for diagnosis and for disease monitoring in PACNS.

NfL is a cytoskeletal protein reflecting neuroaxonal damage and has emerged as a promising biomarker across a variety of neurological disorders, including MS, ALS, and traumatic brain injury (TBI) [17,18,19]. Our findings in patients with PACNS demonstrate markedly elevated NfL levels in both the serum and CSF of patients with aPACNS compared to rPACNS patients and healthy controls, which is indicative of significant neuroaxonal injury driven by acute inflammation and ischemia. The median CSF NfL concentration in aPACNS (4914.58 pg/mL) exceeded the levels that are typically reported in Alzheimer’s disease (1337 pg/mL), dementia with Lewy bodies (1270 pg/mL), and MS (1579 pg/mL). Similarly, the median serum NfL levels (45.77 pg/mL) were higher than those observed in MS (11 pg/mL) or Alzheimer’s disease (19 pg/mL) [20]. These findings suggest that NfL could potentially allow for discriminating PACNS from other neurological diseases. Nonetheless, methodological differences in the NfL measurements between studies can complicate direct comparison and limit the interpretability of absolute NfL values in terms of establishing cut-off values, emphasizing the importance of developing standardized protocols and age-adjusted reference ranges.

Furthermore, while NfL is highly sensitive for detecting neuroaxonal injury, its specificity remains limited, particularly in distinguishing inflammatory from non-inflammatory etiologies. To overcome this limitation, the integration of additional biomarkers may prove valuable. Circulating endothelial cells (CECs), a marker of vascular damage which has previously been shown to be elevated in active PACNS [21], could complement NfL measurement in PACNS by capturing the vascular component of the disease pathogenesis. Combining NfL with CEC measurements in PACNS could thus potentially improve the diagnostic accuracy.

In our analysis, elevated NfL was detectable in 96% of CSF and 70% of serum samples from patients with aPACNS, while none of the rPACNS patients demonstrated elevated NfL levels in either serum or CSF. These observations emphasize NfL’s potential as a sensitive biomarker for distinguishing active from inactive disease. Incorporating NfL measurements into clinical practice could potentially accelerate the diagnosis of disease onset and relapse, facilitating earlier initiation or adaptation of the immunosuppressive regimen, and therefore might be useful for improving patient outcomes.

The relationship between elevated NfL and recent ischemic lesions observed in our correlation analysis highlights an important confounder when evaluating NfL as a diagnostic marker for PACNS. Ischemic stroke frequently occurs in PACNS, complicating the differentiation between inflammatory-driven and ischemia-induced NfL elevations. It is known that NfL levels typically rise acutely following stroke and subsequently decline [22,23,24], emphasizing the need for careful interpretation of NfL elevations in the context of recent ischemic events. Previous studies have reported serum NfL concentrations ranging from 39 pg/mL to 210 pg/mL in stroke patients [25], partially overlapping with the levels observed in our PACNS cohort. In our study, 60% of the aPACNS patients had recently experienced a stroke. However, in our subgroup analysis excluding patients with recent strokes, we confirmed persistently elevated NfL levels (90% in CSF, 50% in serum) in non-stroke PACNS patients, indicating a significant inflammatory contribution to NfL elevation, irrespective of ischemic events. Interestingly, statistical differences in NfL levels persisted among younger patients without recent strokes when compared with both inactive PACNS patients and controls. In contrast, no significant differences were observed in older patients. This is possibly due to age-related increases in baseline NfL levels, diminishing the discriminatory power of NfL, particularly in the serum of older aged PACNS patients. Given that the median age at PACNS diagnosis is around 50 years [26] and the small sample size in this subgroup, this phenomenon’s clinical relevance may be less pronounced. However, this highlights the importance of using age-adjusted reference values.

Notably, patients with biopsy-proven PACNS had lower NfL concentrations than those who were diagnosed radiologically, suggesting distinct disease phenotypes. Biopsy confirmation is more commonly performed in suspected small-vessel variants, which often present with smaller parenchymal lesions compared to medium- or large-vessel variants [27]. In our cohort, only two of the nine patients with biopsy-proven PACNS had recently suffered a stroke. This observation suggests that the lower NfL levels in biopsy-proven cases may partially reflect an interplay between stroke occurrence and the diagnostic approach. Further research should clarify how vessel size involvement and diagnostic methods affect NfL levels, potentially increasing its clinical utility.

As anticipated, we observed a correlation between serum and CSF NfL levels, consistent with prior studies. This supports serum NfL as a viable non-invasive addition for disease monitoring, potentially reducing the reliance on invasive procedures such as lumbar punctures. The implementation of serum NfL measurements could significantly enhance clinical monitoring, providing safer and more cost-effective management strategies. Nonetheless, due to its markedly lower sensitivity compared to CSF, the utility of serum NfL remains limited at this stage. Prospective longitudinal studies are required to explore serum NfL dynamics further and to validate its clinical utility in disease monitoring and therapeutic response assessment.

## 5. Limitations and Future Directions

Our study’s primary limitations include the small sample size, particularly for patients in remission, and the lack of multiple time points for NfL measurements. The rarity of PACNS makes assembling larger cohorts challenging, emphasizing the importance of multicenter collaborations to validate these findings more robustly. Evaluating NfL dynamics over time in the CSF and serum of patients with and without recent ischemic lesions is crucial for distinguishing acute NfL elevations caused by stroke from those driven by inflammatory processes. A prospective follow-up study should also include control groups representing the principal non-vasculitic stroke etiologies.

## 6. Conclusions

Our findings support the clinical utility of NfL in both serum and CSF as a promising biomarker for disease activity and diagnosis in PACNS. The incorporation of NfL into clinical practice could significantly enhance diagnostic precision and improve patient management. Although the correlation between serum and CSF NfL suggests a less invasive option for clinical monitoring, our data highlight that CSF measurements remain more sensitive, and that age-related elevations in baseline NfL, as well as recent ischemic events, can complicate the interpretation of NfL. Standardized protocols and age-adjusted reference values are critical for reliable, broadly applicable NfL assessment in PACNS. In addition, combining NfL measurement with markers of vascular injury, such as CEC, may improve the diagnostic specificity. Prospective, longitudinal, and multicenter studies with PACNS patients in different age groups will be essential to confirm these preliminary observations, refine cut-off thresholds, and evaluate whether NfL-guided surveillance can improve outcomes by prompting earlier, more targeted immunosuppressive interventions.

## Data Availability

The datasets generated during the current study are available from the corresponding author on reasonable request.

## Supplementary Materials

The following supporting information can be downloaded at https://www.mdpi.com/article/10.3390/cells14130966/s1: Table S1: Reference values of NfL; Table S2: Patients with active PACNS; Table S3: Patients with PACNS in remission; Table S4: Age-adjusted model-estimated marginal means; Supplementary Figure S1: Log–log scatterplot of paired neurofilament light-chain (NfL) concentrations measured in CSF (*x*-axis) and serum (*y*-axis) from patients with PACNS. Each symbol represents one individual; colour-coding distinguishes active disease (aPACNS, red triangle, n = 20) from remission (rPACNS, green circles, n = 8). Both axes are shown on a base-10 logarithmic scale. The Spearman correlation (r = 0.93, p < 0.001) was calculated for all PACNS patients with paired values and therefore includes both aPACNS and rPACNS cases. Abbreviations: aPACNS: active PACNS, NfL: Neurofilament-light chain, rPACNS: PACNS in remission.

## Abbreviations

The following abbreviations are used in this manuscript:

ALS	Amyotrophic Lateral Sclerosis
aPACNS	Active PACNS
CECs	Circulating Endothelial Cells
CNS	Central Nervous System
CSF	Cerebrospinal Fluid
DSA	Digital Subtraction Angiography
LSD	Least Significant Difference
MRA	Magnetic Resonance Angiography
MRI	Magnetic Resonance Imaging
MS	Multiple Sclerosis
NfL	Neurofilament Light Chain
OCBs	Oligoclonal Bands
PACNS	Primary Angiitis of the Central Nervous System
rPACNS	PACNS in Remission
SD	Standard Deviation
TBI	Traumatic Brain Injury
