# Supplementary material for: Neurofilament Light Chain in Serum and CSF as a Potential Biomarker for Primary Angiitis of the Central Nervous System
Source: Cells. 2025 Jun 24;14(13):966. doi: 10.3390/cells14130966 (PMC12249180; doi:10.3390/cells14130966)
Supplement: Supplementary file 1 [file cells-14-00966-s001.zip › Supplementary Figure S1 Submission 2.pdf]

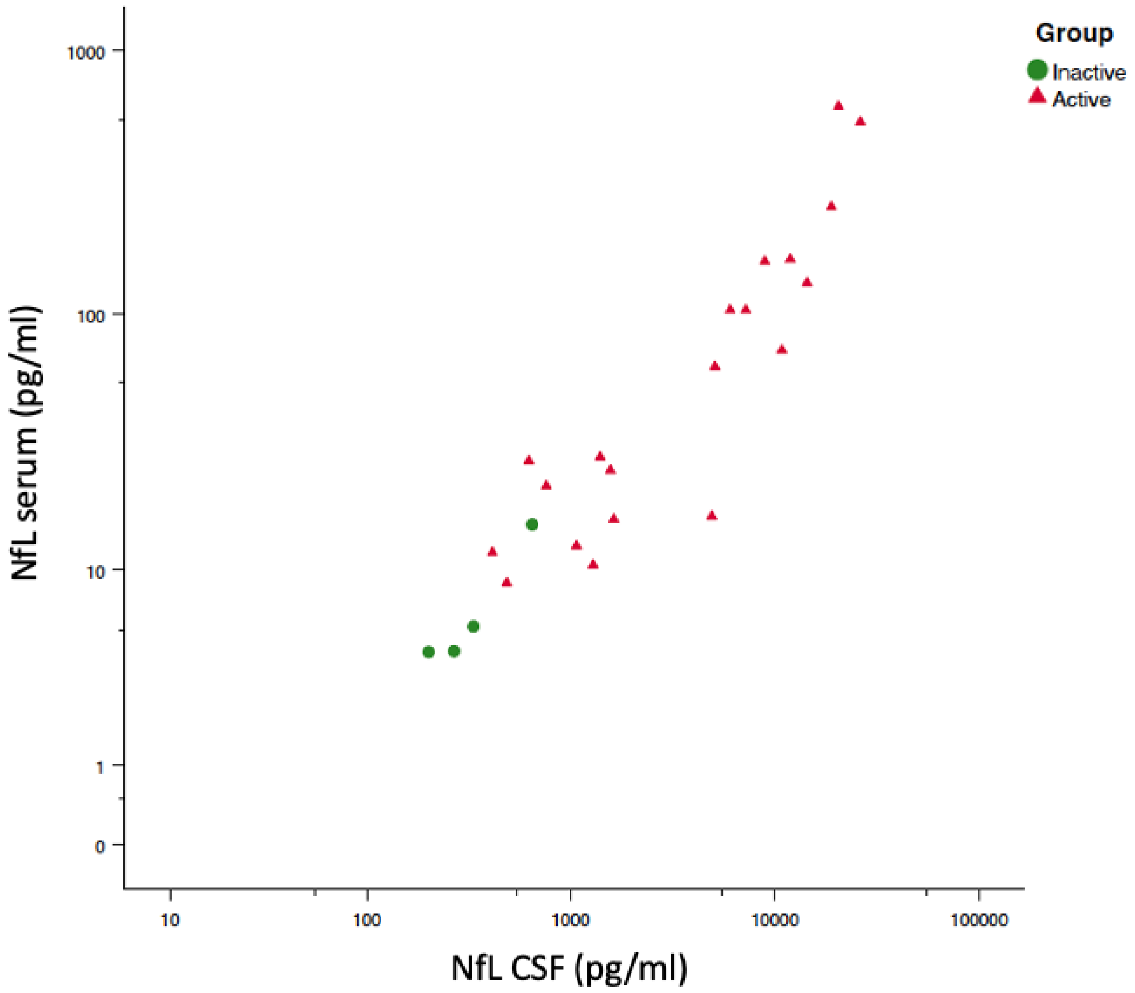

Supplementary Figure S1. Log–log scatterplot of paired neurofilament light-chain (NfL) concentrations measured in CSF (*x*-axis) and serum (*y*-axis) from patients with PACNS. Each symbol represents one individual; colour-coding distinguishes active disease (aPACNS, red triangle, n = 20) from remission (rPACNS, green circles, n = 8). Both axes are shown on a base-10 logarithmic scale. The Spearman correlation (r = 0.93, p < 0.001) was calculated for all PACNS patients with paired values and therefore includes both aPACNS and rPACNS cases. Abbreviations: aPACNS: active PACNS, NfL: Neurofilament-light chain, rPACNS: PACNS in remission.
