# Supplementary material for: Neurofilament Light Chain in Serum and CSF as a Potential Biomarker for Primary Angiitis of the Central Nervous System
Source: Cells. 2025 Jun 24;14(13):966. doi: 10.3390/cells14130966 (PMC12249180; doi:10.3390/cells14130966)
Supplement: Supplementary file 1 [file cells-14-00966-s001.zip › Supplementary Table S1 Submission 2.pdf]

Age	NfL cutoff value
18-50 years	< 12,35 pg/ml
51-60 years	< 20,20 pg/ml
> 61 years:	< 33,61

Supplementary Table S1A: Age adjusted reference values for serum NfL. Abbreviation: NfL: neurofilament light chain.

Age	NfL cutoff value
18-30 years	< 267 pg/ml
31-40 years	< 350 pg/ml
41-50 years	< 436 pg/ml
51-60 years	< 581 pg/ml
> 61 years	< 852 pg/ml

Supplementary Table S1B: Age adjusted reference values for CSF NfL. Abbreviation: NfL: neurofilament light chain.
