# Supplementary material for: Neurofilament Light Chain in Serum and CSF as a Potential Biomarker for Primary Angiitis of the Central Nervous System
Source: Cells. 2025 Jun 24;14(13):966. doi: 10.3390/cells14130966 (PMC12249180; doi:10.3390/cells14130966)
Supplement: Supplementary file 1 [file cells-14-00966-s001.zip › Supplementary Table S2 Submission 2.pdf]

Gender	Age (years)	Diagnosis	Symptoms	MRI/DBI	CSF	DSA/MRA	Biopsy	Disease course	Immunosuppressive treatment
f	35	Suspected PACNS	Headache, hemianopsia	Subacute infarction in the left posterior territory, no contrast enhancement	WBC: 0/3/µl, Protein: 247 mg/l, OCB: 2 isolated bands	Occlusion of the PCA in the P2 segment, stenosis and contrast enhancement of the ICA on both hemispheres	N.p.	Active (onset)	None
f	55	Suspected PACNS, - DD atherosclerosis	Left sided hemiparesis	Multifocal right hemispheric ischemic lesions with contrast enhancement	WBC: 8/3/µl, Protein: 406 mg/l, OCB: neg.	Irregularities and stenosis of the right hemispheric ACA and MCA with contrast enhancement	Neg.	Active (onset)	None
f	33	Suspected PACNS	Hemianopsia, left sided paresthesia	Bihemispheric subacute thalamic ischemic lesions with contrast enhancement, acute ischemic lesion in the right ocipital lobe	WBC: 3/3/µl, Protein: 358 mg/l, OCB: neg.	Progressive stenosis of the BA with contrast enhancement	N.p.	Active (relapse)	Orale cortisone
f	41	Biopsy proven PACNS (lymphocytic PACNS)	Headache, cognitive impairement, left sided hemiparesis	Multifocal FLAIR-hyperintense cortical and subcortical lesions, contrast enhancement of the lesion in the right parietal lobe	WBC: 2/3/µl, Protein: 643 mg/l, OCB: neg.	No pathologies	Positive	Active (onset)	None
m	55	Suspected PACNS	Aphasia, cognitive impairement	Left hemispheric subacute stroke in the basal ganglia with contrast enhancement	WBC: 176/3/µl, Protein: 584 mg/l, OCB: 2 isolated bands, cyto: lymphomonocytic pleocytosis	Stenosis of the PCA on the right, distal MCA stenosis on the left, no contrast enhacenment	N.p.	Active (relapse)	Intravenous cortisone
f	26	Suspected PACNS	Left sided hemiparesis	Multifocal right hemispheric ischemic lesions, no contrast enhancement	WBC: 2/3/µl, Protein: 282 mg/l, OCB: negative, lactate: 2,5 mmol/l	Occlusion/stenosis of the MCA on the right in the M1 segment, no contrast enhacement	N.p.	Active (onset)	None
m	38	Suspected PACNS	Headache, left sided hemiparesis, double vision	No acute/new lesions, older ischemic lesions in the right media territory and the right SCA territory, no contrast enhancement	WBC: 6/3/µl, Protein: 611 mg/l, OCB: negative, lactate: 2,4 mmol/l	Stenosis of the MCA in the M1-Segment with contrast enhacement	Negative	Active (relapse)	Cortisone
f	46	Biopsy proven PACNS	Headache, cognitive impairement, dizziness	Diffuse, symmetrical, radial supra- and infratentorial subcortical lesions with contrast enhancement	WBC: 216/3/µl, Protein: 1273 mg/l, OCB: isolated OCB in CSF and serum, cyto: lymphomonocytic pleocytosis	No pathologies	Positive	Active (onset)	None

m	59	Suspected PACNS	Right sided hemiparesis, headache	Subacute striatic infarctions on both hemispheres with contrast enhancement	WBC: 32/3/µl, Protein: 400 mg/l, intrathekal igA > IgM synthesis, OCB: no OCB, cyto: lymphomonocytic pleocytosis	Irregularities and stenosis of the right hemispheric ACA and MCA with contrast enhancement	N.p.	Active (onset)	None
f	26	Biopsy proven PACNS	Headache, dizziness, transient left sided hemiparesis	No acute/new lesions, no contrast enhancement	WBC: 64/3/µl, Protein: 324 mg/l, OCB: no, cyto: lymphomonocytic pleocytosis	Stenosis and irregularities of the MCA in the M1-Segment, Stenosis of the ACA in the A1-Segment, contrast enhancement of the MCA	Positive	Active (relapse)	None
m	45	Suspected PACNS	Transient left sided hemiparesis	Right hemispheric subacute lesions temporoparietal and frontal with contrast enhancement	WBC: 43/3/µl, Protein: 226 mg/l, intrathecal IgG, IgA >IgM synthesis OCB: pos., cyto: lymphomonocytic pleocytosis	Stenosis of the MCA in the M1/M2 Segment with contrast enhancement	Neg.	Active (onset)	Intravenous cortisone, oral cortisone
m	32	Sususpected PACNS	Cognitive impairement, seizure, transient paresis of the left arm	Multiple bihemispheric infarctions with inhomogenous contrast enhancement and hemorrhagic transformation	WBC: 33/3/µl, Protein: 612 mg/l, OCB: neg. cyto: lymphomonocytic pleocytosis	No pathologies	Neg.	Active (onset)	Oral cortisone
m	39	Suspected PACNS	Aphasia, headache	Subacute infarction in several territories, nodular contrast enhancement parietooccipital	WBC: 214/3/µl, Protein: 709 mg/l, OCB: neg. cyto: lymphocytic pleocytosis	Irregularities of the MCA in the M2-Segment, no contrast enhancement	Neg.	Active (onset)	None
f	58	Biopsy proven PACNS	Transient paresis of the right hand, dysarthria	Multiple supratentorial white matter lesions, no contrast enhancement	WBC: 28/3/µl, Protein: 468 mg/l, OCB: neg. cyto: lymphomonocytic pleocytosis	Multifocal irregularities and stenosis in all territories of the cerebral arteries, no contrast enhancement	Pos.	Active (onset)	None
m	35	Biopsy proven PACNS	Aphasia, cerebellar syndrome, cognitive deficites	New right hemispheric lesions periventricular with affection of the right putamen, multiple older right hemispheric lesions, no contrast enhancement	WBC: 3/3/µl, Protein: 824 mg/l, OCB: neg.	Stenosis of the left PCA in the P3-Segment, no contrast enhancement	Pos.	Active (relapse)	None
m	36	Suspected PACNS	Left sided hemiparesis	Multifocal lesions in the right media territory with contrast enhancement	WBC: 4/3/µl, Protein: 347 mg/l, OCB: neg.	Stenosis of the right MCA in the M1-Segment and the left ICA/MCA with contrast enhancement	N.p.	Active (onset)	None

f	43	Biopsy proven PACNS	Aphasia, right-sided hemiparesis, hemianopsia	T2 hyperintense white matter lesions in both hemispheres, barrier dysfunction on the left hemisphere supra- and periventricular	WBC: 3/3/µl, Protein: 436 mg/l, OCB: pos.	No pathologies	Pos.	Active (relapse)	Azathioprin, Rituximab
f	47	Suspected PACNS	Headache, Aphasia, transient right sided paresis of the leg	New ischemic cortical lesions on the left hemisphere	WBC: 9/3/µl, Protein: 588 mg/l, OCB: neg.	No pathologies	Neg.	Active (relapse)	None
m	74	Biopsy proven PACNS (amyloidangiopathy associated PACNS)	Mild cognitive deficites	New temporal lesions on the right hemisphere, progress of the known lesions, e.g. parietoocipital, no contrast enhancement	WBC: 4/3/µl, Protein: 699 mg/l, OCB: neg.	Contrast enhancement and thickening of the right ICA and in the vetebrobasilar territory, irregularities of the right PCA	Pos.	Active (relapse)	CYC
f	47	Suspected PACNS	Cognitive impairement, residual left-sided hemiparesis, transient diplopia	FLAIR-hyperintense cortical lesions in the media-territory on both hemispheres, no contrast enhancement	WBC: 76/3/µl, Protein: 444 mg/l, OCB: pos., cyto: lymphomonocytic pleocytosis	No pathologies	N.p.	Active (relapse)	None
f	60	Suspected PACNS	Right sided hemiparesis, aphasia	Subacute ischemic lesion in the right ACA territory with contrast enhancement, multiple postischemic lesions in the left hemisphere	WBC: 2/3/µl, Protein: 227 mg/l, OCB: neg.	Irregularities and stenosis of multiple intracranial arteries, contrast enhancement of the MCA with stenosis of the M1-segment	Neg.	Active (onset)	None
m	69	Suspected PACNS	Dysarthria, neglect	Subacute ischemic lesion in the right MCA territory with contrast enhancement, multiple T2-hyperintense white matter lesions	WBC: 0/3/µl, Protein: 522 mg/l, OCB: neg.	Occlusion of the MCA in the M1-segment, contrast enhancement of both MCAs	N.p.	Active (onset)	None
f	65	Suspected PACNS	Headache, hemianopsia	ICB on the right ocipital lobe, multiple T2-hyperintense white matter lesions with contrast enhancement on both hemispheres	WBC: 47/3/µl, Protein: 402 mg/l, OCB: neg., cyto: n.p.	Irregularities of multiple intracranial arteries, thickening of the MCA on both hemispheres, no contrast enhancement	Neg.	Active (onset)	None
m	49	Biopsy proven PACNS (lymphocytic PACNS)	Dyslexia, cognitive deficites	Progressing extense T2-hyperintense white matter lesion on both hemispheres, contrast	WBC: 6/3/µl, Protein: 327 mg/l, OCB: pos.	n.p.	Pos.	Active (onset)	None

				enhancement not assessable due to movement of the patient					
m	47	Biopsy proven PACNS (small-vessel PACNS)	Diplopia, headache	No new lesions, no contrast enhancement	WBC: 120/3/µl, Protein: 575 mg/l, OCB: neg, cyto: n.p.	Stenosis of the left ACA in the A1/A2 segment, stenosis of the left PCA in the P1/2 segment, no contrast enhancement	Pos.	Active (relapse)	None

Supplementary Table S2: Patients with aPACNS. Abbreviations: ACA: anterior cerebral artery, DBI: dark blood imaging, DSA: digital subtraction angiography, f: female, ICA: internal carotid artery, MCA: middle cerebral artery, m: male, MRA: magnetic resonance angiography, MRI: magnetic resonance imaging, MTX: methotrexate, n.p.: not performed, OCB: oligoclonal bands, PCA: posterior cerebral artery, SCA: superior cerebellar artery, WBC: white blood count
