# Supplementary material for: Neurofilament Light Chain in Serum and CSF as a Potential Biomarker for Primary Angiitis of the Central Nervous System
Source: Cells. 2025 Jun 24;14(13):966. doi: 10.3390/cells14130966 (PMC12249180; doi:10.3390/cells14130966)
Supplement: Supplementary file 1 [file cells-14-00966-s001.zip › Supplementary Table S3 Submission 2.pdf]

Gender	Age (years)	Diagnosis	Symptoms	MRI/DBI	CSF	DSA/MRA	Biopsy	Disease course	Immunosuppressive treatment
m	53	Suspected PACNS	No new symptoms, residual cognitive deficites and headache	No new lesions, no contrast enhancement	WBC: 1/3/µl, Protein: 430 mg/l, OCB: neg.	Stenosis of the right MCA in the M1-segment and the ACA in the A1 and A2 segment, no contrast enhancement	N.p.	Inactive	None
m	61	Suspected medium-vessel PACNS	No new symptoms, residual cognitive deficites and left sided hemiparesis	No new lesions, contrast enhancement	WBC: 0/3/µl, Protein: 465 mg/l, OCB: neg.	No pathologies	N.p.	Inactive	MTX 15 mg s.c.
f	30	Suspected monophasic PACNS	Headache, dizziness	Multiple small FLAIR-hyperintense lesions periventricular on both hemispheres, DBI: neg.	WBC: 5/3/µl, Protein: 693 mg/l, OCB: identical bands	Chronic occlusion of the left ICA, no contrast enhancement	N.p.	Inactive	None
f	46	Suspected medium-vessel PACNS	No new symptoms	N.p.	WBC: 4/3/µl, Protein: 267 mg/l, OCB: neg.	N.p.	N.p.	Inactive	MMF
m	72	Biopsy proven PACNS	No new symptoms, residual neglect	N.p.	N.p.	N.p.	Positive	Inactive	Rituximab
f	33	Suspected PACNS	No new symptoms	N.p.	N.p.	N.p.	N.p.	Inactive	None
f	39	Biopsy proven PACNS (granulomatous PACNS)	Headache, dizziness	N.p.	N.p.	N.p.	Positive	Inactive	Infliximab 500 mg, MTX 15 mg s.c.
m	71	Biopsy proven PACNS (necrotizing PACNS)	No new symptoms, residual cognitive deficits	N.p.	N.p.	N.p.	Positive	Inactive	None

Supplementary Table S3: Patients with PACNS in remission. Abbreviations: ACA: anterior cerebral artery, CSF: cerebrospinal fluid, DBI: dark blood imaging, DSA: digital subtraction angiography, f: female, ICA: internal carotid artery, MCA: middle cerebral artery, m: male, MRA: magnetic resonance angiography, MRI: magnetic resonance imaging, MTX: methotrexate, n.p.: not performed, OCB: oligoclonal bands, WBC: white blood count
