# Supplementary material for: Neurofilament Light Chain in Serum and CSF as a Potential Biomarker for Primary Angiitis of the Central Nervous System
Source: Cells. 2025 Jun 24;14(13):966. doi: 10.3390/cells14130966 (PMC12249180; doi:10.3390/cells14130966)
Supplement: Supplementary file 1 [file cells-14-00966-s001.zip › Supplementary Table S4 Submission 2.pdf]

Age (years)	Group	NfL Serum (pg/ml)				NfL CSF (pg/ml)			
		Effects: Group, p < 0.001; Age, p< 0.001; Gender, p = 0.701; Group x Age, p = 0.049; adj. R^2 = 0.531				Effects: Group, p < 0.001; Age, p = 0.047; Gender, p = 0.09; Group x Age, p = 0.038; adj. R^2 = 0.677			
		Mean		95% Confidence Interval		Mean		95% Confidence Interval	
				Lower Bound	Upper Bound			Lower Bound	Upper Bound
30	Inactive	4,4	a	3,2	6,6	241,0	a	108,1	616,3
	Active	20,9	b	11,3	44,9	1460,2	b	744,5	3067,2
	Control	4,7	a	4,5	4,9	180,7	a	156,5	209,4
70	Inactive	17,1	a	9,6	35,2	485,0	ab	164,7	1782,4
	Active	23,4	a	12,1	54,3	1514,5	b	739,6	3352,0
	Control	12,6	a	11,3	14,3	692,6	a	529,2	910,6

Supplementary Table S4. Age-adjusted model-estimated marginal means with 95% confidence intervals, back-transformed to the original scale (pg/mL), for patients without recent stroke. In younger individuals, NfL levels are significantly higher in aPACNS patients compared to rPACNS patients and healthy controls. With increasing age, group differences diminish. Significant differences persist longer in CSF than in serum. Within each age group and column, means sharing the same letter are not significantly different based on the least significant difference (LSD) test.
